# Data indicating temperature response of Ti–6Al–4V thin-walled structure during its additive manufacture via Laser Engineered Net Shaping

**DOI:** 10.1016/j.dib.2016.02.084

**Published:** 2016-03-09

**Authors:** Garrett J. Marshall, Scott M. Thompson, Nima Shamsaei

**Affiliations:** a Department of Mechanical Engineering, Mississippi State University, PO Box 9552, 39762 MS, USA; b Center for Advanced Vehicular Systems (CAVS), Mississippi State University, PO Box 5405, 39762 MS, USA

**Keywords:** Additive manufacturing, Laser Engineered Net Shaping (LENS), Directed energy deposition, Infrared thermography, Process monitoring, Thermal imaging, Melt pool, Heat affected zone, Heat transfer

## Abstract

An OPTOMEC Laser Engineered Net Shaping (LENS^™^) 750 system was retrofitted with a melt pool pyrometer and in-chamber infrared (IR) camera for nondestructive thermal inspection of the blown-powder, direct laser deposition (DLD) process. Data indicative of temperature and heat transfer within the melt pool and heat affected zone atop a thin-walled structure of Ti–6Al–4V during its additive manufacture are provided. Melt pool temperature data were collected via the dual-wavelength pyrometer while the dynamic, bulk part temperature distribution was collected using the IR camera. Such data are provided in Comma Separated Values (CSV) file format, containing a 752×480 matrix and a 320×240 matrix of temperatures corresponding to individual pixels of the pyrometer and IR camera, respectively. The IR camera and pyrometer temperature data are provided in blackbody-calibrated, raw forms. Provided thermal data can aid in generating and refining process-property-performance relationships between laser manufacturing and its fabricated materials.

**Specifications Table**

**Table:** 

Subject area	*Mechanical Engineering*
More specific subject area	*Additive Manufacturing*
Type of data	*Comma Separated Values (CSV) and Audio Video Interleaved (AVI) files*
How data was acquired	*Infrared camera (Sierra-Olympic Technologies, Inc. Viento320*^*™*^*SL-V1-36NO)*
*Camera control and image acquisition software v. 1.7.14 (DRS Technologies, Inc.)*
*Dual-wavelength pyrometer (Stratonics, Inc.)*
*Data acquisition system (Measurement Computing*^*™*^*USB-1208FS) routed from pyrometer to computer*
*Stratonics ThermaViz® software*
Data format	*Raw*
Experimental factors	*Plasma Rotating Electrode Process (PREP) Ti-6Al-4V powder, in its as-received form (Phelly Materials, Inc), was utilized. The Ti–6Al–4V substrate was sandblasted, cleaned with soap, rinsed with alcohol, and dried via compressed air. Thermal cameras were pre-calibrated to a black body source, though no emissivity correction has been applied to the IR data. A post-processing, IR temperature correction method is described in detail in*[1].
Experimental features	*An OPTOMEC LENS*^*™*^*750 system*[2], *equipped with a 1 kW Nd:YAG laser (IPG), was utilized to fabricate a thin-walled structure from Ti–6Al–4V powder. Manufacturing was performed in an argon-purged environment with the following parameters: 290* *W laser power, 12.7* *mm/s scan rate, and 0.32* *g/s powder feed rate. A dual-wavelength pyrometer*[3], [4]*and infrared (IR) camera monitored the thermal response of the melt pool and bulk part, respectively*.
Data source location	*Center for Advanced Vehicular Systems (CAVS), Starkville, Mississippi, USA*
Data accessibility	*All data have been deposited to the Data in Brief Dataverse.*

**Value of the data**

• Data provide a means to generate and validate numerical and/or analytical models for laser-based additive manufacturing processes, as well as other manufacturing methods (e.g. welding and cladding).
• Data allow for the quantification of temperature and heat transfer related to directed energy deposition and other additive and/or laser-based manufacturing methods.
• Data allow for quantification of the laser-induced, powder-fed melt pool wetting characteristics, morphology, cooling rates, stability, and more. This enables a better physical understanding of the complex transport phenomena within and around the melt pool, which can support research into pore formation and solidification.
• Data allow one to ascertain process-property relationships for additively-manufactured parts; providing a means to relate observed microstructural features with experimentally measured temperature distributions.
• Experimental results may be used for developing or enhancing manufacturing process control and/or thermal monitoring techniques, including: IR camera calibration based on angle and emissivity, process parameter selection for effective deposition, and determination of material optical properties such as emissivity and absorptivity.

## 1 Data

Thermal images captured via dual-wavelength pyrometry and infrared (IR) thermography are provided as Comma Separated Values (CSV) files. Each file displays a grid of temperature values (degrees Celsius) corresponding to pixels of either the pyrometer or IR camera. Files have been named with respect to time and position of the melt pool (*t-**time**_x-**location**_y-**location**_z-**location_**layer**#**.csv)* unless no melt pool was present (*t-**time**_ChangingZ**#*** and *zChanging_noise**#*** for the IR camera and pyrometer respectively). The IR temperature data has been provided in degrees Celsius, but these specific measurements are not corrected for the emissivity of the Ti–6Al–4V during its manufacture. Select files collected from both the pyrometer and IR camera were used to create video depicting real-time thermal behavior of melt pool and bulk part, respectively. All data, including raw files and video, have been deposited to the Data in Brief Dataverse.

## 2 Experimental design, materials and methods

### 2.1 General equipment and experimental set-up

**Fig. 1:**
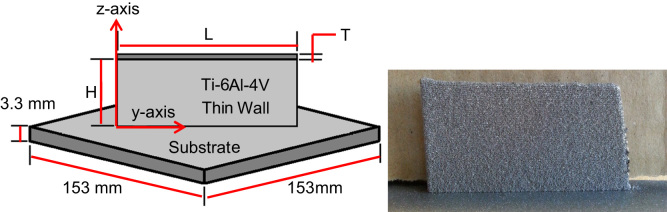
As-built Ti-6Al-4V thin wall: dimensions (left) and photograph (right).

An OPTOMEC LENS^™^ 750 system equipped with a 1 kW Nd:YAG laser (IPG) was utilized for additively-manufacturing a thin wall from Ti–6Al–4V powder. The LENS™ system consisted of four angled nozzles (Ø=1.016 mm) to allow for the continuous injection of powder into the laser beam and melt pool during manufacture. The Ti–6Al–4V powder (Phelly Materials, Inc.) was spherical in shape as created via Plasma Rotating Electrode Processing (PREP). The powder possessed a size distribution between 44 μm and 149 μm (−100+325 mesh). Powder was utilized in its as-received condition from manufacturer, i.e., powder was not recycled from a previous fabrication. The Ti–6AL–4V substrate possessed dimensions of 153×153×3.3 mm^3^ and was mounted atop a copper alloy 110 plate (153×153×6.4 mm^3^). The copper plate was utilized as a spacer allowing the Ti–6Al–4V substrate to be adequately clamped to the Computer Numerical Controlled (CNC) build platform during manufacture. The thin wall was fabricated to meet set dimensions shown in Table 1; however, actual (measured) dimensions differed slightly. Fig. 1 provides a dimensioned schematic and photograph of the thin wall. Parameters for the fabrication process were set as follows: 290 W laser power, 12.7 mm/s scan rate, and 0.32 g/s powder feed rate. The build chamber was purged continuously with room-temperature argon at approximately 4 L/min. Argon was fed into the system through a port between the four co-axial nozzles.

The utilized build pattern is now described. Upon activation of the laser, the CNC stage moves right relative to the deposition head (powder injector with laser) for a distance of 50.8 mm, whereupon the laser is deactivated and the first layer of part manufacture is complete. After depositing the single track/layer, the deposition head increments upward by 0.508 mm, and the CNC stage returns to the starting x-and y-location of the previous layer and begins the process anew until a total of 60 layers have been deposited. Once activated, the laser stayed in place for 20 μs prior to CNC stage motion. While the laser was activated, the scan rate was 12.7 mm/s; while off, i.e. repositioning to begin a new track, the scan rate was set to 25.4 mm/s which began 2000 μs after laser deactivation. Acceleration and deceleration of the melt pool were set to 423.3 mm/s^2^; that is to say, when the build stage was at zero velocity or approaching zero velocity, it accelerated/decelerated at the prescribed value until the desired velocity (25.4 mm/s or 0 mm/s) was achieved.

Since the thin wall consisted of individual layers with single-track thickness, no Computer-Aided Design (CAD) file was utilized. Instead, the numeric control file was customized in which no wall thickness value was required. Final part thickness was a consequence of laser power, scan speed, and powder feed rate parameters (see Table 1).

**Fig. 2:**
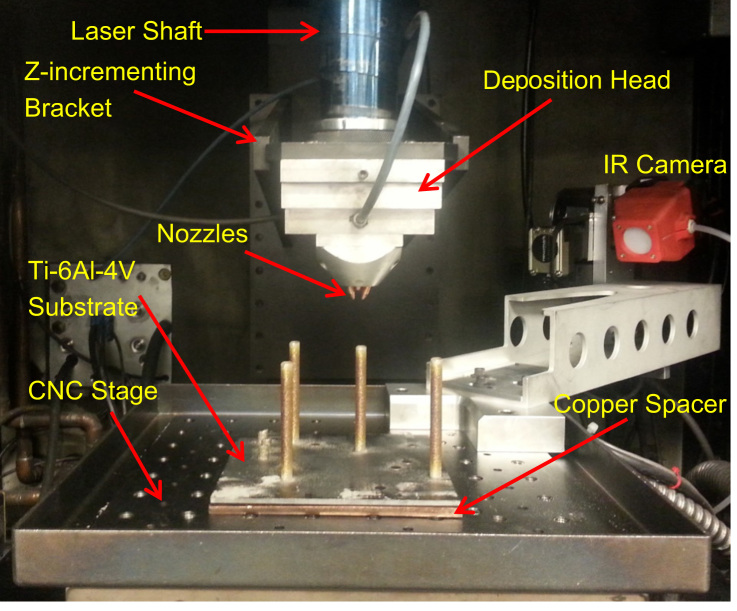
Photograph of inside the LENS™ chamber (window cover removed) with IR camera on custom mounting bracket, Ti–6Al–4V substrate, Ti–6Al–4V cylindrical rods, CNC stage, and deposition head consisting of nozzles and laser shaft. A ‘z-incrementing bracket’, which is connected to the laser shaft, is also shown in photograph for reference. Cylindrical specimens shown in photograph were in the chamber at the time of photograph and are unrelated and inconsequential to the presented data [1].

The distance between the nozzle tips and the top of the substrate (i.e. standoff distance), at the beginning of process, was measured to be approximately 6.7 mm. At the completion of the build, the distance between the nozzles and the top of the fabricated part was measured to be approximately 10.2 mm. The setup for the current experiment was similar to that shown in Fig. 2. Height-wise displacement of deposition head (in the z-direction) was set to move in increments of 0.508 mm between layers for a total of 60 layers (a process lasting seven minutes, thirty-seven seconds). Oxygen content within the chamber was 16.2 ppm and 14.9 ppm at the beginning and end of the process, respectively.

### 2.2 Camera configurations

A dual-wavelength pyrometer (Stratonics, Inc.) and an IR camera (Sierra-Olympic Technologies, Inc. Viento320) were used to capture temperature of the melt pool and part during manufacture, respectively. The pyrometer was mounted above the OPTOMEC LENS^™^ 750 machine, outside of the inert environment of the chamber and aligned so as to view down the laser shaft via a series of three, broadband metallic mirrors (Newport 20D20ER.1). Specifications of interest for the pyrometer are provided in Table 2. Exposure time was set to 2.0274 ms and the pixel clock was set to 5 MHz. The nominal image collection rate of the pyrometer was approximately 6.4 Hz.

**Fig. 3:**
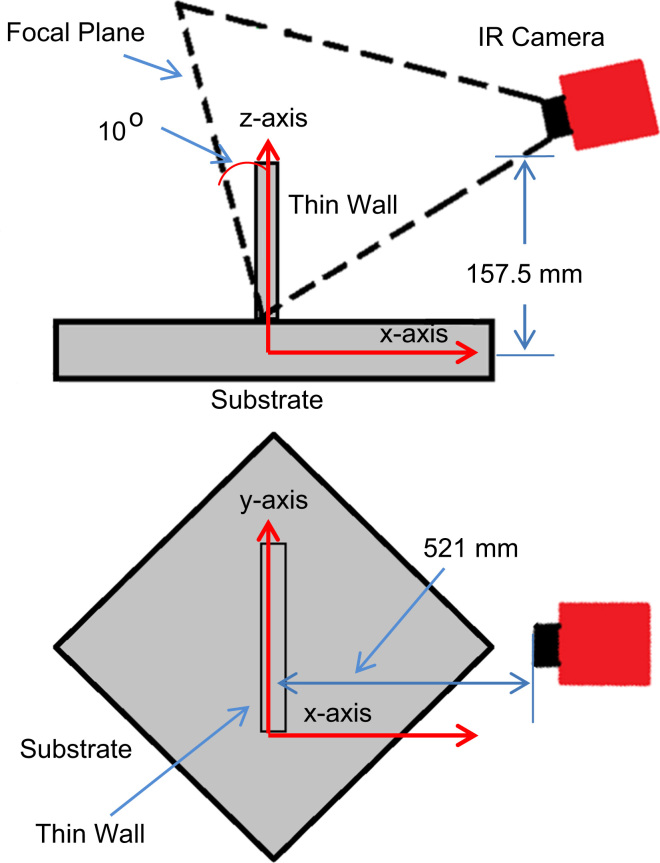
Side view (top) and aerial view (bottom) of IR camera and its orientation with respect to substrate and thin wall within the build chamber [1].

Fig. 3 provides a side and top view of the IR camera within the LENS^™^ build chamber. The IR camera was oriented at approximately 45° with respect to the sides of the CNC stage, and as seen in Fig. 3, was tilted in such a way that the focal plane was rotated 10° from a line normal to the substrate. The thin wall was constructed at an orientation such that one of its sides was fully in-view by the IR camera. The IR camera was custom-mounted to the CNC stage within the build chamber to allow the thin-walled structure to appear static in the field-of-view and be located within the IR focal plane. Data provided from the IR camera do not contain exact temperatures as it is difficult to relate IR signals to absolute temperatures, especially during the phase change process. Specifications of interest for the IR camera are provided in Table 2. The nominal image collection rate of the IR camera was approximately 12.58 Hz.

**Fig. 4:**
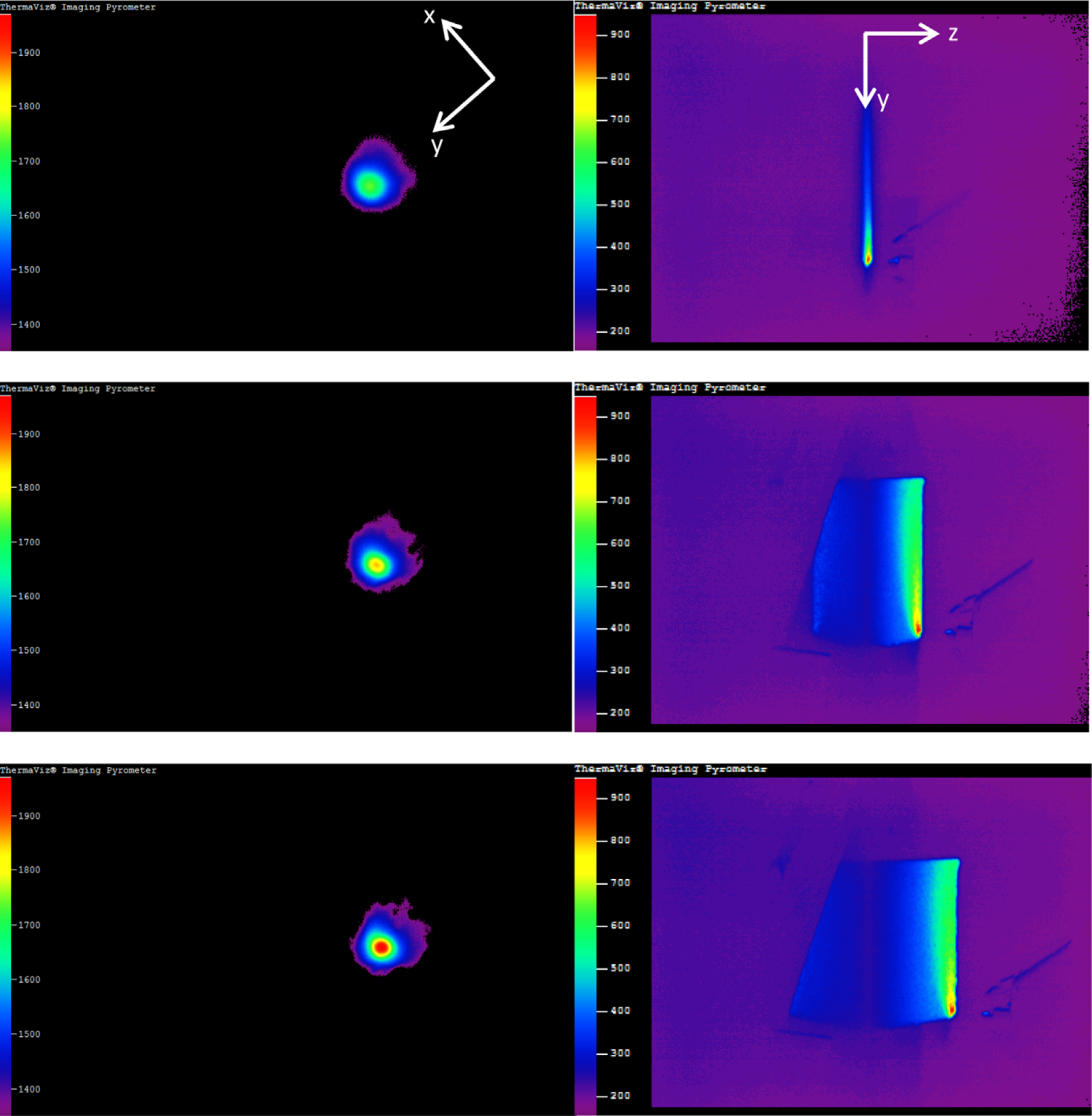
Pyrometer (left) and IR (right) images collected at the beginning, middle and end of manufacturing Ti-6Al-4V thin wall via LENS^TM^. A coordinate frame has been overlaid on the top images for reference.

File names are displayed as *t-**time**_x-**location**_y-l**ocation**_z-**location_**layer**#**.csv*; this naming scheme describes the relative time an image was taken, as well as the relative melt pool location. An example file name is: t2p468_x0_y31p34_z0_layer1. Numerical time and location values follow their respective variable names. The ‘p’ seen in the name represents a decimal point and has been added to keep the files temporally sequenced when organized by file-folder managers. Some files do not contain active melt pools, corresponding to times when the laser was off, i.e. when the CNC stage was returning to its start position before depositing the next layer. These files are labeled by noting that the z-height is changing in the file name: *t-**time**_ChangingZ**#**.csv*, where ***#*** represents the original file number and has only been kept to aid in file sorting. Since the z-height starts at 0 mm, the final z-height is 30.09 mm as opposed to the input part height, 30.48 mm. Fig. 4 provides actual thermal images taken from the build of the Ti–6Al–4V wall. Images depicting deposition during the first, middle and final layers are shown. Note that raw IR images are tilted 90° to the right when captured. No attempt has been made to adjust this in the CSV files. As a consequence, the laser appears to be traveling from top to bottom of the image instead of left to right as reality would have it.

## Figures and Tables

**Table 1:** Set and measured dimensions of Ti–6Al–4V thin-walled structure.

	**Length (L)**	**Height (H)**	**Thickness (T)**
**Set dimension values**	50.8 mm	30.48 mm	N/A
**Measured dimension values**	47.81 mm@top	27.56 mm	1.78 mm
51.78 mm@base

**Table 2:** Pyrometer and IR camera specifications.

**Pyrometer Specifications**	
Detector type	CMOS
Array size	752×480
Pixel pitch	6.45 μm
Temperature range	1000–2500 °C

**IR Camera Specifications**	
Detector type	Uncooled VOx Microbolometer
Array size	320×240
Pixel pitch	17 μm
Response range	8–14 μm
NEdT @f/1.0	<50 mK
